# Cultural humility: treating the patient, not the illness

**DOI:** 10.3402/meo.v21.30908

**Published:** 2016-02-03

**Authors:** Sunila J. Prasad, Pooja Nair, Karishma Gadhvi, Ishani Barai, Hiba Saleem Danish, Aaron B. Philip

**Affiliations:** Faculty of Medicine, Imperial College London, London, UK

Patient populations across the world are becoming increasingly diverse, introducing a variety of health behaviours that are influenced by a patient's cultural background. *Tomorrow's Doctors* guidelines state that all qualified doctors must respect patients ‘without prejudice’, irrespective of ‘diversity of background and opportunity, language, culture and way of life’ (1). Are medical students currently being fully supported to acquire this fundamental skill?

A suggested definition of culturally competent care assumes that healthcare providers can ‘learn a quantifiable set of attitudes and communication skills’ that will allow them to work effectively within the cultural context of the patients they come across (2). However, the broad nature of cultural competency limits its integration into an already intense medical curriculum (3). So, how can developments in medical education overcome this challenge? It can be done by promoting cultural humility.

In the medical context, cultural humility may be defined as a process of being aware of how people's culture can impact their health behaviours and in turn using this awareness to cultivate sensitive approaches in treating patients (4).

Unlike cultural competency, there is no specific end point to cultural humility as we are not being asked to demonstrate a ‘quantifiable set of attitudes’. This concept is a continual process, one that requires self-reflection and self-critique. Developing cultural humility in itself is a prerequisite to cultural competency. It does so by forming a foundation for students to consider possible power imbalances that may arise between a doctor and patient when cultural differences may have an impact on the potential clinical outcome for the patient. Subsequently, the student may be encouraged to develop approaches and skills that could contribute to a harmonious dynamic of the doctor–patient relationship (5). Patient care is individualised as we take time to consider a patient's personal beliefs rather than attempting to place them under a cultural label. Developing cultural humility will therefore allow students to appreciate someone's culture as a dynamic entity.

Drawing upon the philosophy of Daoism, which is based on the concept of humility leading to the attainment of knowledge, Chang et al. argue that cultural humility can greatly increase the student's receptiveness to learn about their own attitudes (5). Chang et al. further describes the concept of cultural humility in which the elements of self-questioning, immersion into an individual patient's point of view, active listening, and flexibility all serve to confront and address cultural biases or assumptions a student may hold. In clinical practice, lack of awareness of our cultural perceptions introduces the risk of subconscious imposition of our beliefs during patient interactions (6).

To facilitate this skill amongst medical students, engagement with the humanities, for example, literature, art, or poetry, may be encouraged. Reading a book that explores another culture may enable us to reflect on our own reactions to the content of the book, rather than simply learning about another culture's practices (7). Cultural humility is a concept that admittedly does not easily lend itself to generic methods of assessment producing pass or fail results. Methods of assessment should therefore in some way complement the dynamic nature of developing the skill, for example, engaging in reflective writing or participating in group discussions with peers after reading a book that explores cultural issues. Reflective pieces of writing can consequently be discussed with a communication skills tutor, for example, who might also play a role in facilitating peer group discussions. A level of self-assessment may also be suitable, for example, through questionnaires that explore a student's ideas about different cultures (8). If such activities are weaved into the medical school curriculum, for example, by integrating them into existing communication skills teaching sessions, the potential strain on time and resources may be alleviated.

Investigating methods of teaching and assessing cultural humility have been explored in small residency programmes in the United States, for example, by Juarez et al., which demonstrate positive outcomes with regard to patient satisfaction (8). Whilst this letter has proposed promoting cultural humility in the medical school system, it also seeks to highlight that further evidence needs to be collected in order to assess the strength of the impact of cultural humility on patient encounters and the long-term effects on a student's professionalism in a culturally diverse patient setting.

Increased awareness of cultural humility and its integration into the medical student curriculum would have universal benefits for medical students and patient care. Further work on fully establishing the utility of culture humility within medical education should be welcomed. Ultimately, it presents itself as an ethos in medical education that requires further promotion, as it can facilitate the development of culturally sensitive doctors who deliver a standard of care patients deserve.

*Sunila J. Prasad* Faculty of Medicine, Imperial College London London, UK Email: sunila.prasad11@imperial.ac.uk *Pooja Nair* Faculty of Medicine, Imperial College London London, UK *Karishma Gadhvi* Faculty of Medicine, Imperial College London London, UK *Ishani Barai* Faculty of Medicine, Imperial College London London, UK *Hiba Saleem Danish* Faculty of Medicine, Imperial College London London, UK *Aaron B. Philip* Faculty of Medicine, Imperial College London London, UK
